# Hibernation Impairs Odor Discrimination – Implications for Alzheimer’s Disease

**DOI:** 10.3389/fnana.2019.00069

**Published:** 2019-07-16

**Authors:** Torsten Bullmann, Emily Feneberg, Tanja Petra Kretzschmann, Vera Ogunlade, Max Holzer, Thomas Arendt

**Affiliations:** ^1^Department of Molecular and Cellular Mechanisms of Neurodegeneration, Paul Flechsig Institute for Brain Research, University of Leipzig, Leipzig, Germany; ^2^Department of Neuropathology, University of Leipzig, Leipzig, Germany

**Keywords:** Alzheimer’s disease, microtubule associated protein tau, protein phosphorylation, spines, torpor, memory

## Abstract

Reversible formation of PHF-like phosphorylated tau, an early feature of Alzheimer’s disease (AD) was previously shown to occur in torpor during hibernation in the Golden hamster (Syrian hamster, *Mesocricetus auratus*). Here, we tackled the question to what extent hibernating Golden hamsters can serve as a model for the early stage of AD. During early AD, anosmia, the loss of olfactory function, is a common and typical feature. We, thus, investigated tau phosphorylation, synaptic plasticity and behavioral physiology of the olfactory system during hibernation. Tau was phosphorylated on several AD-relevant epitopes, and distribution of PHF-like phosphorylated tau in the olfactory bulb was quite similar to what is seen in AD. Tau phosphorylation was not associated with a destabilization of microtubules and did not lead to fibril formation. Previously, we observed a transient spine reduction in pyramidal cells in the hippocampus, which is correlated with the distribution of phosphorylated tau. Here we show that granule cells in the olfactory bulb are devoid of phosphorylated tau and maintain their spines number during torpor. No reduction of synaptic proteins was observed. However, hibernation did impair the recall performance in a two-odor discrimination task. We conclude that hibernation is associated with a specific olfactory memory deficit, which might not be attributed to the formation of PHF-like phosphorylated tau within the olfactory bulb. We discuss a possible involvement of modulatory input provided by cholinergic neurons in the basal forebrain, which are affected by hibernation.

## Introduction

The microtubule-associated protein tau promotes polymerization (Weingarten et al., 1975), stabilizes existing microtubules (Drechsel et al., 1992), organizes them into bundles (Elie et al., 2015) and mediates interaction of microtubules with filamentous actin (Biswas and Kalil, 2017; Cabrales Fontela et al., 2017). Phosphorylation of serine and threonine residues modulates these function and leads to a detachment of tau from microtubules. Whereas dephosphorylated tau is present in the axon, its phosphorylation results in its accumulation in the somatodendritic and subsynaptic compartment (Lindwall and Cole, 1984; Biernat et al., 1993; Buée et al., 2000; Morris et al., 2011). In neurodegenerative diseases commonly referred to as “tauopathies” the accumulation of hyper-phosphorylated tau leads to the formation of paired helical filaments (PHF), the structural subunits of neurofibrillary tangles and other aggregates (Avila, 2000; Shahani and Brandt, 2002; Brandt et al., 2005; Avila et al., 2008; Goedert and Spillantini, 2011). Long before the formation of PHFs and subsequent neuronal degeneration, the presence of hyperphosphorylated tau in the post-synaptic compartments leads to the deterioration of synaptic plasticity and cognitive decline (Yoshiyama et al., 2007; Hoover et al., 2010; Ittner et al., 2010; Morris et al., 2011). This is supported by the presence of tau aggregates in dendritic spines (Blazquez-Llorca et al., 2011) at very early stages of Alzheimer’s disease.

Paired helical filaments-like hyperphosphorylation of tau is not restricted to neurodegeneration, but occurs under physiologically controlled conditions such as hibernation (Arendt et al., 2003; Härtig et al., 2007; Stieler et al., 2009, 2011; Avila et al., 2012; Bullmann et al., 2016). Subsequent anatomical and biochemical studies on different hibernating species revealed a profound synaptic regression in cortex, hippocampus and thalamus (Popov and Bocharova, 1992; Arendt et al., 2003; von der Ohe et al., 2006, 2007). The likely consequence of synaptic changes is an impairment of cognitive functions, such as learning and memory, that has been studied using different learning paradigms in various species (McNamara and Riedesel, 1973; Millesi et al., 2001; Zhao et al., 2004; Clemens et al., 2009; Bullmann et al., 2016); for a review see (Arendt and Bullmann, 2013). However, the outcome of these studies revealed a dependency on species-specific preparedness (Bullmann et al., 2016). Under laboratory conditions, we observed a preservation of spatial memory following hibernation in golden hamsters (Bullmann et al., 2016), while an impairment was observed in European ground squirrels (Millesi et al., 2001). In their natural habitat, however, this two species employ different overwintering strategies and favor specific modalities to be preserved. The Golden hamster is strictly solitary and digs rather complex burrows to store the horded food for the hibernation period (Gattermann et al., 2001). In contrast, groups of European ground squirrel cuddle in a single nest, relying on body fat instead of stockpiled food as energy resource (Hut and Scharff, 1998).

Therefore, European ground squirrels must recognize conspecifics while the impairment of spatial memory (Millesi et al., 2001) does not impact survival. Contrary, the Golden hamster needs to preserve spatial memory but not necessarily social recognition. For regulating social interactions hamsters almost exclusively use olfactory cues in Johnston (1990), Petrulis (2009). Both main olfactory system (MOS) and the accessory or vomeronasal system (AOS) process social cues (Meredith, 1991). Both systems have separate sensory neurons and segregated representations in the olfactory bulbs. The sensory neurons of the MOS detect primarily volatile chemicals and make synapses with dendrites of mitral/tufted cell within the glomeruli of main olfactory bulb. Processing of olfactory information from the periphery to central brain structures occurs along a neuronal network, consisting of several well defined relays (Barkai and Saar, 2001; Wilson et al., 2006). This includes layer II pyramidal cells in the piriform cortex (Knafo et al., 2001) as well as hippocampal CA3 pyramidal cells (Hess et al., 1995), CA1 pyramidal cells (Knafo et al., 2004) and the prefrontal cortex (Alvarez and Eichenbaum, 2002). Already within the olfactory bulb, the lateral inhibition (Lledo and Lagier, 2006) by the reciprocal connections between mitral and granule cells increase discrimination of odors (Mori et al., 1999). Rodents have a highly effective olfactory modality and show rapid acquisition and sustained recall of odor discrimination (Slotnick and Katz, 1974; Schellinck et al., 2001; Friedrich, 2006).

Therefore, we combined behavioral, biochemical and morphological analyses to test whether hibernation induced phosphorylation of tau in the main olfactory bulb is associated with synapse regression and affects odor discrimination and recognition memory. Previously, we presented preliminary results in abstract form at a conference (Bullmann et al., 2009).

## Materials and Methods

### Animals

Male and female Golden hamsters (Syrian hamsters, *Mesocricetus auratus*) purchased from Harlan Winkelmann GmbH (Borchen, Germany) were bred and housed at the Medizinisch Technisches Zentrum of the Medical Faculty of the University of Leipzig. Animals had free access to food and water and were maintained on an artificial 12:12 h light-dark cycle under conditions of constant temperature (22°C) and humidity. All experimental procedures on animals were carried out in accordance with the European Council Directive of 24 November 1986 (86/609/EEC) and had been approved by the local authorities. All efforts were made to minimize the number of animals used and their suffering.

A total of 76 animals were subjected to hibernation conditions (Ueda and Ibuka, 1995) as described before (Bullmann et al., 2016). Briefly, they were maintained in an animal incubator (8:16 h light-dark cycle; 23–26°C) for four to 8 weeks and then in the cold room (4:20 h light-dark cycle; 5–7°C). General locomotor activity was monitored with custom build infrared detectors mounted on top of each cage allowing the discrimination between euthermic phases and torpor. Hibernating animals show torpor phases (over 24 h of inactivity) whereas non-hibernating euthermic animals did not. For sampling hamsters were allocated to one of five groups depending on their time of inactivity after an arousal episode as torpor early (TE; 8 h of inactivity) or torpor late (TL; 36–48 h of inactivity), according to the time after induction of arousal as arousal early (AE; 2.5 h) and arousal late (AL, 24–36 h), and those did not show torpor as euthermic (EU).

Sample preparation was performed as previously described (Bullmann et al., 2016): Animals were killed by CO_2_, followed by transcardial perfusion with physiological saline followed by 4% paraformaldehyde and 0.1% glutaraldehyde in phosphate buffered saline (PBS). After immersion for 2 days in 30% sucrose in PBS, brains where cut into 30 μm thick coronal sections and stored at 4°C in PBS with 0.01% sodium azide added as a preservative. For western blotting brains were dissected, samples were shock frozen in liquid nitrogen, and stored at -80°C.

### Human Cases

For each case (Table 1), one olfactory bulb and a block containing the hippocampus and entorhinal cortex were fixed for 2 weeks in neutral formalin and immersed in 30% sucrose in phosphate buffered saline (PBS) with sodium azide added as a preservative. Afterward 30 μm thick frozen sections were cut and stored in PBS with sodium azide at 4°C.

**Table 1:** Human cases.

Case number	Diagnosis	Death
2–97	control	multiple organ failure
39–96	Alzheimer’s disease	multiple organ failure
40–96	Alzheimer’s disease	hemothorax leftside
3–88	Alzheimer’s disease	Unknown


All cases were neuropathologically assessed for neurofibrillary tangle stage (Braak and Braak, 1991; Braak et al., 2006), for amyloid beta/amyloid plaque score (Thal et al., 2002) and for neuritic plaque score according to CERAD (Gearing et al., 1995).

The diagnosis of Alzheimer’s disease (AD) was made on the basis of both clinical and neuropathological evidence according to the criteria of the International Working Group (IWG) for New Research Criteria for the diagnosis of AD in the revision of 2014 (IWG-2) (Dubois et al., 2014), the NIA-AA diagnostic criteria in the revision of 2011 (Albert et al., 2011) and the NIA-AA guidelines for the neuropathological assessment of AD (Mirra et al., 1991).

### Immunohistochemistry

Immunohistochemical processing was performed as described before (Arendt et al., 2003; Bullmann et al., 2007, 2010, 2016; Härtig et al., 2007). Briefly, free-floating sections were rinsed in PBS, and incubated 30 min in 0.3% hydrogen peroxide in PBS (phosphate buffered saline). As from now TBS (Tris buffered saline: 100 mM NaCl, 50 mM Tris/HCl pH 7.4) was used with Triton X-100 added at 0.01% for rinsing and 0.1% for antibody incubation. Sections were rinsed, incubated 60 min in 5% normal donkey serum (Amersham), and primary antibodies where applied in 1% normal donkey serum overnight (see Table 2). For immunofluorescent labeling, sections were rinsed, incubated for 2 h with appropriate carbocyanine 2 (Cy2) and carbocyanine 3 (Cy3) conjugated secondary antibodies. The selected donkey antibodies recognizing goat, rabbit or mouse IgG, 1:1000 (Jackson Immuno Research, West Grove, PA, United States) were affinity-purified and showed minimal cross-reactivity. Sections were then rinsed, mounted, dehydrated, cleared, and coverslipped with Entellan. For immunoperoxidase labeling, sections were rinsed, incubated for 2 h with the appropriate biotin-conjugated secondary antibody. The selected donkey antibodies recognizing rabbit or mouse IgG, 1:1000 (Jackson Immuno Research, West Grove, PA, United States) were affinity-purified and showed minimal cross-reactivity. Afterward, sections were rinsed, incubated for 45 min with ExtrAvidin (1:1,000; Sigma), rinsed, and developed with nickel-enhanced diaminobenzidine, mounted, dehydrated, cleared, and coverslipped with Entellan.

**Table 2:** List of antibodies used for immunohistochemistry.

Name [epitope]	Host, type	Dilution	Source; designation
AT8 [pS202/pT205]	mouse, mc	1:1,000	Pierce; MN1020
DCX	goat, pc	1:500	Santa Cruz; sc-8066
CB	rabbit, pc	1:5,000	Swant; CD-28a
ChAT	goat, pc	1:100	Chemicon; AB144
TH	goat, pc	1:200	Santa Cruz; sc-7847


*Legend: pc, polyclonal; mc, monoclonal; DCX, doublecortin; CB, calbindin; ChAT, choline acetyl transferase; TH, tyrosine hydroxylase; mc, monoclonal; pc, polyclonal.*

### Western Blots

Western blots were performed as previously described (Arendt et al., 2003; Härtig et al., 2005; Stieler et al., 2011; Bullmann et al., 2016). Briefly, tissue samples were homogenized in tenfold volume of ice cold buffer A (20 mM Tris–HCL, pH 7.2; 150 mM NaCl; 2 mM MgCl2; 2 mM EDTA; 2 mM EGTA; 5 mM NaF; 1 mM Na3VO4; 5 % glycerol) supplemented with protease inhibitors (1 mM PMSF; 1 μg/ml leupeptin; Complete protease inhibitor cocktail from Roche). Homogenates were centrifuged (50,000 × *g*, 30 min, 4°C), and supernatants (“cytosolic fraction”) were transferred into new tubes. Pellets were suspended in buffer B (buffer A + 0.5% Triton X-100) supplemented with protease inhibitors (see above), centrifuged again and supernatants (“membrane fraction”) were transferred into new tubes.

Protein samples in Laemmli buffer of 20 μg protein per lane were separated on a 10% polyacrylamide gel by SDS-PAGE and transferred to a PVDF membrane. After rinsing with TBS-Tw (100 mM NaCl, 50 mM Tris/HCl pH 7.4, 0.05% Tween 20) and blocking (1% w/v bovine serum albumin in TBS-Tw) they were incubated overnight with primary antibodies (Table 3) in blocking buffer. After rinsing they were incubated 2 h with the appropriate horseradish peroxidase conjugated secondary antibodies (GE Healthcare; 1:10,000 sheep-anti-Mouse IgG, NA931V; 1:10,000 donkey-anti-Rabbit IgG, NA934V) and rinsed. Immunoreactivity was determined by enhanced chemoluminescence (0.23 mg/ml Luminol; 0.1 mg/ml p-coumaric acid and 0.6 mg/ml sodium perborate in 0.1 M Tris–HCL, pH 8.6), acquired with a Kodak Image Station 2000R, and quantified by densitometric analysis using TINA (version 2.09 g, 1993, raytest Isotopenmeßgeräte GmbH). The expression level was set to 1 for the euthermic group, by dividing the arbitrary fluorescence units with the median of the respective euthermic group. Simple measurements were shown as dots and a line connects the median for each group (see Figure 2). For statistic testing these expression levels were log10-transformed in order to obtain normality of their distribution. In case of the six phosphorylation-dependent antibodies a MANOVA was performed before assessing the results for each individual antibody by separate ANOVA and Tukey’s HSD test. Preliminary experiments had shown that the blot and detection efficiency decreases toward the edges of the blot. In order to prevent a bias, we therefore applied the protein samples cyclically according to their state (EU>TE>TL>AE>AL) from the left to the right. Furthermore, the spatial difference in the blot and detection efficiency was then controlled in ANOVA by using the position on the blot (the cycle number WBCYCLE) as a blocking factor. If ANOVA showed significant influence of the physiological state, the fit was used for the *post hoc* Tukey’s HSD test for comparison between the different states.

**Table 3:** List of primary antibodies used on immunoblots of protein fractions.

Name [epitope]	Host, type	Dilution	Source; designation	Fraction
anti-human tau [243-441]^∗^	rabbit, pc	1:2,000	Dako; A0024	A
AT270 [pT181]	mouse, mc	1:500	Pierce; MN1050	A
AT8 [pS202/pT205]	mouse, mc	1:500	Pierce; MN1020	A
AT100 [pT212/pS214/pT217]	mouse, mc	1:500	Pierce; MN1060	A
AT180 [pT231/pS235]	mouse, mc	1:500	Pierce; MN1040	A
PHF1 [pS396/pS404]	mouse, mc	1:2,000	Peter Davies	A
Tau-1 [S202/T205]	mouse, mc	1:1,000	Chemicon, MAB3420	A
VGluT1	guinea pig, pc	1:1,000	Synaptic Systems; 135–304	B
PSD-95	mouse, mc	1:500	Millipore; 05-494	B
gephyrin	rabbit, pc	1:500	Synaptic Systems; 147-003	B
synaptophysin	mouse, mc	1:10,000	DAKO; clone SY38; M776	B
α-tubulin	mouse, mc	1:1,000	Sigma; clone DM1A; T6199	B
acetylated tubulin	mouse, mc	1:4,000	Sigma; clone 6-11B-1; T6793	B
β-actin^∗∗^	mouse, mc	1:20,000	Sigma; A5316	A, B


*Legend: pc, polyclonal; mc, monoclonal; A, cytosolic fraction; B, detergent soluble (“membrane”) fraction. ^∗^According to the datasheet the immunogen was recombinant human tau protein expressed in *E. coli*, corresponding to the C-terminal part (amino acids 243–441) containing the four repeated sequences involved in microtubule binding. The epitope does not include the additional microtubule-binding domain of the 4R tau, because the antibody recognizes all tau isoforms. ^∗∗^Antibody was applied after stripping as loading control for each immunoblot of each fraction.*

### Antibodies

For summary see Tables 1, 2. Tau phosphorylation was studied by phosphorylation-dependent tau antibodies in hibernating animals including European ground squirrels [*Spermophilus parryii*: (Arendt et al., 2003)], Golden hamster [*Mesocricetus auratus*: (Härtig et al., 2005; Stieler et al., 2011; Bullmann et al., 2016)], arctic ground squirrel [*Spermophilus citellus*: (Stieler et al., 2011)] and black bear [*Ursus americanus*: (Stieler et al., 2011)]. Again, these antibodies will be used to determine the phosphorylation of Golden hamster tau. Microtubule stability was assessed by immunohistochemical detection of α-tubulin and its acetylation, because it is known that tubulin monomers with this modification are enriched in stable microtubules (Janke and Montagnac, 2017). Furthermore, transfection with microtubule-associated proteins MAP1B, MAP2 or tau increase both microtubule stability and tubulin acetylation (Takemura et al., 1992). Synaptic proteins were measured by antibodies directed against synaptophysin (present in all pre-synapses) (Calhoun et al., 1996; Tarsa and Goda, 2002; Strijkstra et al., 2003), gephyrin (present in GABAergic post-synapses) (Peden et al., 2008) as well as VGluT1 (glutamatergic pre-synapses) (Härtig et al., 2003; Graziano et al., 2008) and PSD95 (glutamatergic post-synapses) (Mondragón-Rodríguez et al., 2012; Nair et al., 2013).

### Golgi Impregnation and Spine Measurements

Golgi impregnation of neurons was performed according to the Golgi-Bubenaite method (Bubenaite, 1929) as previously described (Bullmann et al., 2016). Briefly, brains were fixed by transcardial perfusion with 4% paraformaldehyde and 0.1% glutaraldehyde in phosphate buffered saline (PBS), olfactory bulb samples were immersed for one day in 2.5% potassium dichromate, one day in 3.5% potassium dichromate at 37°C, washed in 2% silver nitrate and incubated in the dark for 2 days in 2% silver nitrate at 37°C. Afterward samples were dehydrated and embedded in celloidin. Sections of 100 μm thickness were cut, mounted onto glass slides and coverslipped with Canada balsam.

In the main olfactory bulb, only GABAergic granule cells possess spines, which are part of the bidirectional dendrodendritic synapses with the Glutamatergic mitral cells. Blind measurements were performed on the spine density of single granule cell dendrites within the external plexiform layer of the olfactory bulb; short straight sections within one focal plane selected and traced using the Neurolucida^TM^ system (MicroBrightField, Inc.) at highest magnification (100×, immersion lens). The spine density (spines/μm) was log10-transformed in order to obtain normality of their distribution. The influence of physiological state on spine density was assessed by a nested ANOVA, where the single measurements were nested within each animal.

### Olfactory Discrimination Task

Olfactory discrimination task was performed similar as described for mice (Schellinck, 2001). The test apparatus was constructed from a large housing cage divided into three equal compartments by two pieces of acrylic that had openings of 6 cm diameter that could be closed by sliding doors made of acrylic. Therefore, animals could be placed in the middle compartment and after opening of the doors, they could freely move from one compartment to another. Phenyl acetate (Sigma Aldrich, “rose”) and Linalool (Sigma Aldrich, “lemon”) where diluted to a concentration of 15% in mineral oil (Sigma Aldrich). The odors were the same as in the original procedure (Schellinck et al., 2001). Linalool is a naturally occurring terpene alcohol found in many flowers and spice plants, whereas phenyl acetate is an aromatic fatty acid metabolite of phenylalanine naturally occurring in mammals. They have been selected for having a different chemical structure, possess distinct smells and bind to different subsets of olfactory receptors and activate olfactory receptor neurons of the clusters B and C, respectively (Mori et al., 2006). A volume of 50 μl of either lemon or rose odor were pipetted onto filter paper, placed in petri dishes with holes in the top, and buried with pine chips each in one of the side chambers. The animal was placed in the middle chamber and the sliding doors were opened after 5 min of habituation. The behavior was then recorded for 30 min by a small IR-camera module (191710-62, Conrad Electronics, Germany) connected to a recorder and analyzed off-line. During testing, the three chamber apparatus was covered by a mesh top and was placed under a fume hood.

One week prior training animals were habituated to the test procedure. During the initial habituation we decided to not present any of the two odors, because after such initial presentation, the animal recognizes both them as an unrewarded cue and the subsequent training will elicit a weaker conditioned response. This is sometimes referred to as negative priming (Wagner, 1981). Animals were placed in new cages with fresh beddings at lights on, and 2 h after lights off habituation to the test procedure started as described above, but without odors in the petri dishes. Later animals were placed back into their home cages with free access to food and water.

After 2 months under short day conditions, 2 weeks before transfer to the cold room, animals were trained. One half of the hamsters received rose paired with food reward (three sunflower seeds) and lemon alone; the other half received lemon paired with food reward and rose alone. The choice of sunflower over the original sugar reward (Schellinck et al., 2001)seed was motivated by a study on suitable food rewards in learning and memory test in hamsters (Shettleworth, 1978) and we have used them successfully in a previous behavioral study of memory in hibernating hamsters (Bullmann et al., 2016). Animals were placed in cages without food at lights on, training started at 2 h after lights off and an hour after training animals were allowed free access to food. In two subsequent weeks, subjects were trained on four consecutive days and received four trials per day, which consisted of alternating trials of rose and lemon, resulting in a total of 16 trials for each CS+ and CS-. As described in the original procedure, in each trial only one odor was presented to the animal (see Supplementary Figure S2).

After training animals were transfer to the cold room and continuously monitored for hibernation behavior (see section 2.1). After half of the animals had entered hibernation, all animals were removed from the cold room and maintained under short day conditions with 25°C room temperature and free access to food and water for 2 days before testing. A total of 35 hamsters were tested, 17 hibernating and 18 non-hibernating. After final testing, all animals were transferred to the cold room, and approximately 90% entered hibernation afterward.

**FIGURE 1:**
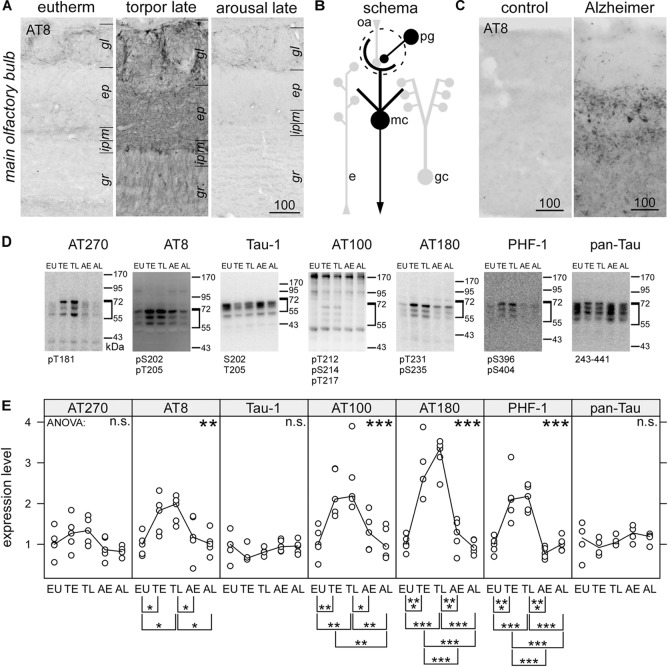
Immunohistochemical detection of phospho-tau by the monoclonal antibody AT8in the olfactory bulb of hamsters during hibernation **(A)** and humans **(C)**. For the human cases, higher magnification images can be found in the Supplementary Figure S1. Legend: gl, glomerular layer; ep, external plexiform layer; m, mitral cell layer; gr, granule cell; lm, stratum lacunosum moleculare; r, stratum radiatum; p, pyramidal cell layer; o, stratum oriens. Scale bars in μm. A schematic description of the distribution of phosphorylated tau is shown for olfactory bulb **(B)**. Structures containing phosphorylated tau are drawn black whereas those devoid of phosphorylated tau are depicted in light gray. Within the olfactory bulb, olfactory receptor cell axons (oa) are forming synapses to the apical dendritic tuft of mitral cells, which project outside the olfactory bulb. Activity of periglomerular cells (pg), lateral inhibition by granule cells (gc) and efferent modulatory input (e) influence this transmission. Phosphorylation of tau protein in the olfactory bulb was compared in euthermic animals (EU), early torpor (ET), late torpor (TL), and after early arousal (AE) and late arousal (AL). Immunoblots **(D)** were probed for the presence of specific PHF-like tau-phospho-epitopes (AT 270; AT8; AT 100; AT180; PHF-1), for tau, unphosphorylated at Ser198, Ser199, and Ser202 (Tau-1) and total tau (pan-tau). ANOVA showed significant differences after quantification **(E)**for AT8 (*p* = 0.017), AT100 (*p* = 0.00032), AT180 (*p* = 2.5e-08), and PHF-1 (*p* = 6.5e-08), but not for AT270 (*p* = 0.096), Tau-1 (*p* = 0.34), and pan-Tau (*p* = 0.21). The antibody epitope is indicated below each blot. Inside the diagrams the following significance code for *p*-values for ANOVA are shown: 0 < “^∗∗∗^” < 0.001 < “^∗∗^” < 0.01 < “^∗^” 0.05 < “n.s.”. The significance codes for *p*-values for the *post hoc* Tukey’s HSD test are shown below the diagrams.

The behavior in the test chamber was measured using the timestamp from the recorded video. The preference for CS+ over CS- (conditioned stimulus with and without food reward, respectively) odor was measured by converting the time spend in the side-chambers to a preference index *P*. *P* was calculated (Kaut et al., 2003) according to the following formula: $P=\frac{x-y}{x+y}$, where x is the amount of time spent in the CS+ chamber, and y is the amount of time spent in the CS- chamber. An index of P = +1 indicates a perfect preference for CS+, an index of P = -1 indicates a perfect preference for CS- whereas an index of 0 reflect chance performance. This preference index was used for the box-whisker plot in Figure 1C.

Additionally, we assessed the motivational state by measuring the exploratory behavior. From the total time spend in the side-chambers during the 30 min test period, an exploratory index *E* was calculated by the following formula: $E=\frac{x+y}{x+y+z}$, where z is the amount of time spent in the middle chamber. An index of 0 reflects that the hamster did not leave the starting chamber, whereas an higher index indicates more exploratory behavior. The preference index and exploratory index were used for the box-whisker plots in Figure 4.

Before the analysis of variance (ANOVA), both indices were Logit-transformed: $LP=\log\left(\frac{P+1}{P-1}\right)=\log\left(\frac{x}{y}\right)$ and $LE=\log\left(\frac{E}{1-E}\right)=\log\left(\frac{x+y}{z}\right)$. This transformation then yields normally distributed data *LP* and *LE* for the relative quantities *P* and *E*, which presumably show a binomial distribution. We assumed that the animals might show preference not only for the conditioned over the unconditioned stimulus, but also for one of the odors or one of the side chambers. Furthermore, the performance might also depend on subtle differences during the training of the four cohorts. In this case a randomized block design should be used (Festing and Altman, 2002). Therefore, the ANOVA for *LP* included the factor HIBERNATION and the three blocking factors ODOR, SIDE, and COHORT. HIBERNATION encoded whether the animal showed at least one torpor episode (yes or no). ODOR and SIDE indicated the odor (rose or lemon) used as the CS+ and the side chamber, which contained the CS+ during the test (left or right). COHORT was a categorical variable with four levels. For the *LE* the *P*-values for the difference were taken from the ANOVA with COHORT as the only blocking factor. The raw data used for the statistical analysis of the two odor discrimination task can be found in Supplementary Table S1.

### Software for Data, Statistics, Diagrams, Figures

Data were organized and stored using Excel and OpenOffice. R^1^ (R Development Core Team, 2011) was used for statistics and with the R package lattice^2^ (Sarkar, 2008) also for diagrams. Final figures were arranged using Canvas X (Deneba Systems). The following significance code for *p*-values were used: 0 < “^∗∗∗^” < 0.001 < “^∗∗^” < 0.01 < “^∗^”0.05 < “n.s.”.

## Results

### Phosphorylation of Tau

Both the olfactory bulb and hippocampus have been implicated in the formation and consolidation of olfactory memory as “peripheral” and “central” relays along the pathway for processing olfactory information (Hess et al., 1995; Lledo and Lagier, 2006). While phosphorylation of tau in the hippocampus of hibernating hamsters was documented recently (Stieler et al., 2011; Bullmann et al., 2016), it has not been assessed in the olfactory bulb before.

**FIGURE 2:**
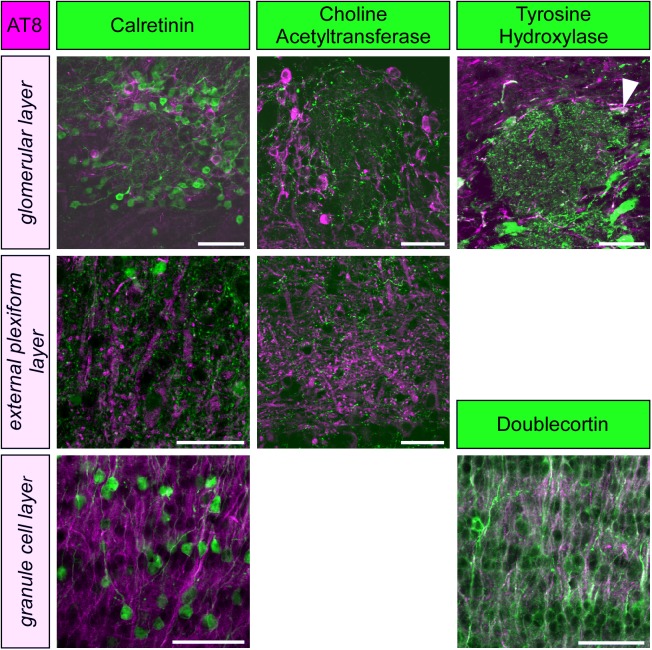
Phospho-tau, detected by the monoclonal antibody AT8 is not co-localized with markers for periglomerular neurons (calretinin, choline acetyltransferase, tyrosine hydroxylase) or granule cells (calretinin, doublecortin) within the olfactory bulb, except for a few tyrosine hydroxylase containing periglomerular neurons (arrowhead). Scale bars: 50 μm.

Using Western blots and immunocytochemistry we observed a profound tau phosphorylation in the olfactory bulb (Figure 1, 2). Western blots of olfactory bulb samples were probed with the following antibodies recognizing different epitopes consisting of phosphorylated serine (pS) and threonine (pT) residues or a combination of several epitopes (Figure 1D): AT270 (pT181), AT8 (pS202+pT205), AT180 (pT231+pS235), PHF-1 (pS396+pS404), and AT100 (pT212+pS214+pT217). The majority of antibodies showed a significant increase in phosphorylation at their respective epitopes during torpor, which was fully reversible after arousal (Figure 1E). Accordingly, a slightly retarded gel migration was observed during torpor using the pan-tau antibody (Figure 1D, rightmost panel). Conversely, dephosphorylation of tau protein after arousal was revealed by monoclonal antibody tau-1, which binds if both S202 and T205 are unphosphorylated.

The epitope pS202+pT205, recognized by the monoclonal antibody AT8, is also found at very early, pre-tangle stages of Alzheimer’s disease (Braak and Braak, 1991). When we mapped the distribution of PHF-like hyperphosphorylated tau by immunohistochemistry using the AT8 antibody we observed an intense staining in the human olfactory bulb (Figure 1C) in cases with Alzheimer’s disease (*N* = 3) but not in control the case (*N* = 1).

Increased immunoreactivity for AT8 during torpor has previously also been found in both obligatory and facultative hibernators (Arendt et al., 2003; Härtig et al., 2007; Bullmann et al., 2016; Stieler et al., 2009, 2011). Within the olfactory bulb of torpid hamsters, a particularly strong labeling was seen in periglomerular cells and the neurites within the glomeruli (Figure 1A). Somewhat weaker reactivity was present in cell bodies of the mitral cell layer and in neurites within the external plexiform layer and the granule cell layer (summarized in Figure 1B). After arousal, immunoreactivity for AT8 vanished rapidly and returned to levels seen in euthermic animals.

To further specify the cellular identity of cells containing PHF-like phosphorylated tau, we performed double labeling with molecular markers of granule cells, periglomerular neurons and cholinergic efferent projections. Phosphorylated tau did not co-localize with any of these markers, and only a small fraction of dopaminergic periglomerular neurons contained phosphorylated tau (Figure 2). Therefore, it can be concluded that phosphorylated tau is mainly present in cell bodies, dendrites and axons of mitral cells.

### Spines Density and Synaptic Proteins

**FIGURE 3:**
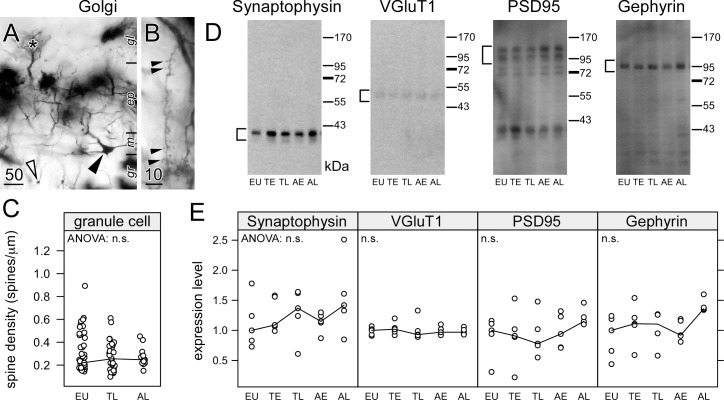
Hibernation does not lead to synaptic regression in the dendrites of granule cells in the olfactory bulb. Hemi brains were impregnated by the Golgi-Bubenaite method, embedded in celloidin and sectioned at 100 μm. In low magnification, granule cell bodies (open arrow) as well as cell bodies (closed arrow) and dendritic tufts of mitral cells within glomeruli (star) **(A)** are visible. Single granule cell dendrites in the external plexiform layer of the olfactory bulb were identified by their morphology and short segments showing numerous spines **(B)** were traced. Spine density **(C)** remained unchanged on granule cell dendrites (ANOVA: *p* = 0.61). Synaptic proteins in the olfactory bulb were compared in euthermic animals (EU), early torpor (ET), late torpor (TL), and after early arousal (AE) and late arousal (AL). Immunoblots **(D)** were reacted for the presence of synaptophysin, VGluT1, PSD95 and gephyrin. ANOVA showed no significant (*p* > 0.05, n.s.) variation after quantification **(E)** for each of them. Scale bars in μm.

Previously, apical dendrites hippocampal CA3 pyramidal neurons were affected much stronger by the accumulation of phosphorylated tau than basal dendrites, and this was correlated with a larger spine regression (Bullmann et al., 2016). Therefore, we analyzed the spine density on the dendrites of granule cells, which do not show accumulation of phosphorylated tau. Olfactory bulb granule cell dendrites were identified by their morphology and localization within the external plexiform layer (Figure 3A). Reconstructions were made from branches containing at least ten large spines (Figure 3B). Consistent with the absence of phosphorylated tau in granule cell dendrites, their spine density remained unaffected during hibernation (Figure 3C, left panel).

Granule cell spines are associated with glutamatergic synapses from mitral to granule cells, but also with reciprocal GABAergic synapses from granule to mitral cells. Theses dendrodendritic synapses are the majority of synapses within the olfactory bulb. Synaptic proteins present in GABAergic synapses (gephyrin), glutamatergic synapses (VGluT1, PSD95) or synapses in general (synaptophysin) were quantified using western blots (Figure 3D). Consistent with the spine measurements, no changes were observed in the amount of synaptic proteins during the hibernation cycle (Figure 3E).

### Olfactory Memory and Motivation

**FIGURE 4:**
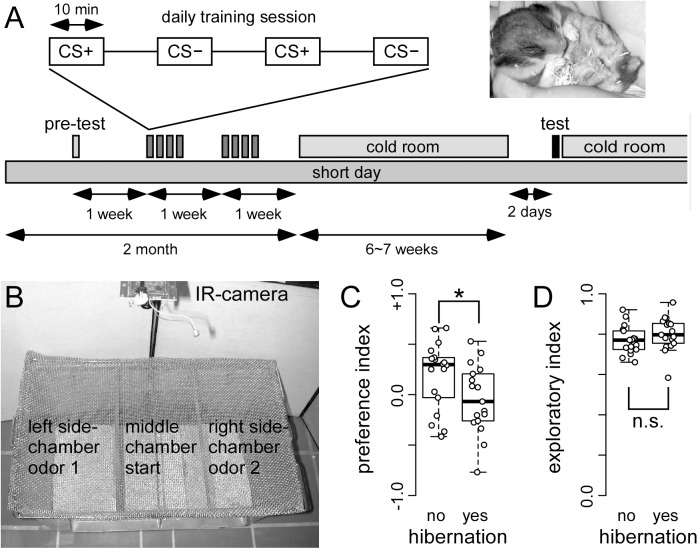
Hibernating hamster fail in a two odor discrimination task **(A)** On each training day, food deprived hamster received trials in which one odor (CS+) was paired with food reward (sunflower seed) and another odor (CS–) was paired with no reward. After a total of 16 of each CS+ and CS– pairings, hamsters were transferred to the cold room. When 50% of the cohort were hibernating (see photo insert), they were removed from the cold room, and tested the other day. A total of 17 and 18 hibernating and non-hibernating hamsters were tested, respectively. The test chamber **(B)** was placed under a fume hood. After being placed in the middle chamber, the hamster could choose to walk and explore the side chambers. These side-chambers were equipped with odor pots used during the training of CS+ and CS–. The behavioral response was recorded using a IR camera. The preference index **(C)** showed that non-hibernating hamster spend more time. This difference was significant (^∗^) as indicated by ANOVA (*p* = 0.027424) in the side-chamber with the CS+ odor. This preference was not observed in hamsters that did hibernate. In contrast, hibernation did not affect the exploration index **(D)** (ANOVA: *p* = 0.2190). The entire hamster did not stay in the middle chamber, but spend most of their time in the side-chambers.

First, we analyzed whether hibernation affects memory in Golden hamsters using an olfactory discrimination task, which allows to reliably assess olfactory learning and memory (Figure 4). During the training phase, one odor (CS+) was paired with a food reward while another odor (CS-) remained unrewarded. After several weeks in the cold, animals were retested for retention. We removed any systematic bias of odor preference by randomizing the odor cue paired with the food reward and during testing half of the animals were presented with the rewarded odor on the left side, for the other half it was presented at the right side. Furthermore, we removed any confounding effect of any group preference for any odor by using it as a blocking factor in the ANOVA. The results show no preference for rose or lemon in hamsters (Table 4).

**Table 4:** Analysis of variance for the olfactory discrimination task.

Measurement	Factor	Df	*F*-value	p
**Preference**	**Hibernation**	**1**	**5.415**	**0.0274**
	*Odor*	*1*	*1.483*	*0.2333*
	*Side*	*1*	*8.049*	*0.0084*
	*Cohort*	*1*	*0.916*	*0.4457*
	Residuals	28		
**Exploration**	**Hibernation**	**1**	**1.576**	**0.2190**
	*Cohort*	*3*	*0.625*	*0.6045*
	Residuals	30		


*We assumed that the choice of odor associated with the reward, placement of the rewarded odor during testing as well as the training cohort could affect the animal behavior. Therefore all behavioral experiments were performed using a randomized block design in order to control for these known sources of variability during ANOVA. We were only interested in the influence of hibernation on the preference for the rewarded odor and exploratory activity (shown in bold letters), with any influence of the nuisance factors removed (shown in italics). Legend: Df, degrees of freedom; p, probability value or significance.*

Non-hibernating animals showed significantly higher preference for the rewarded (CS+) than for the unrewarded (CS-) odor (Figure 4C; *p* = 0.0274), while hibernating hamsters showed no preference. This indicates that non-hibernating animals remembered the odor, which was associated with food reward while animals gone through hibernation did not.

In order to test whether these behavioral changes might potentially be attributed to changes in motivation, we assessed the exploratory behavior of both hibernating and non-hibernating animals. To this end, the time was measured that animals spend in the side chambers of the test apparatus instead of remaining in the middle chamber. No differences were obtained between hibernating and non-hibernating animals (Figure 4D, *p* = 0.2190). This indicates that motivation is not different between non-hibernating animals and animals previously gone through hibernation.

### Stability of Microtubules

**FIGURE 5:**
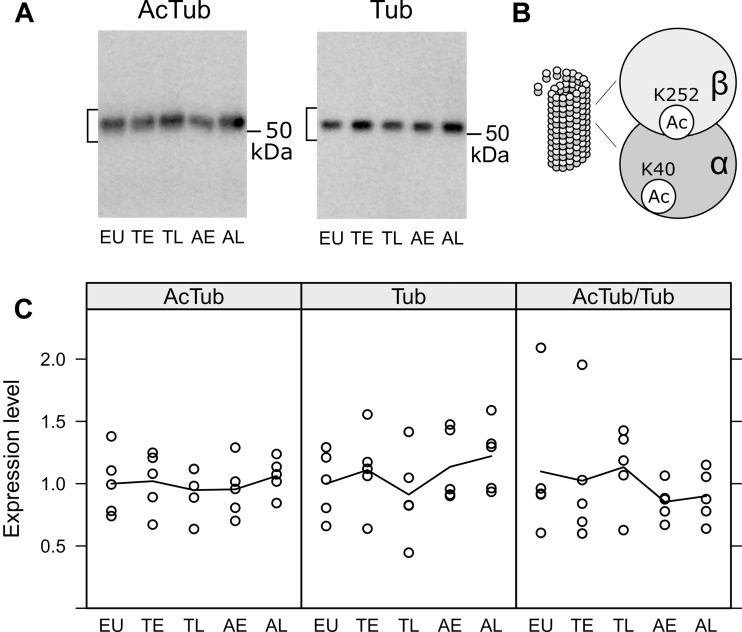
Acetylation of tubulin in the olfactory bulb was compared in euthermic animals (EU), early torpor (ET), late torpor (TL), and after early arousal (AE) and late arousal (AL). Immunoblots **(A)** were probed for acetylated tubulin (AcTub) and alpha tubulin (Tub). Stable microtubules are enriched in acetylated tubulin monomers **(B)**. The densitometric quantification of both as well as the ratio of AcTub/Tub are shown in the diagrams below **(C)**. ANOVA showed no significant differences (*p* = 0.915, 0.548 and 0.759 for AcTub, Tub, and AcTub/Tub), indicating no change in microtubule stability during the course of hibernation.

Next, we analyzed the effects of hibernation on microtubule stabilization, the canonical function of tau protein. During the course of hibernation, there were no obvious changes in the acetylated Tubulin monomers (AcTub) compared to the total amount of alpha-Tubulin (Tub; see Figure 5). This indicates, that microtubule stability might largely remain unaffected during torpor.

## Discussion

In the present study, we combined behavioral, biochemical and morphological analyses to investigate whether hibernation induced phosphorylation of tau in the main olfactory bulb is associated with synaptic regression and a loss of long-term memory for discrimination of odors.

### Phosphorylated Tau in the Olfactory Bulb

Paired helical filaments-like hyperphosphorylation of tau is a typical feature of AD, however, it also occurs under physiologically controlled conditions such as hibernation (Arendt et al., 2003; Härtig et al., 2007; Stieler et al., 2009, 2011; Avila et al., 2012; Bullmann et al., 2016). Previous reports have shown, that both temperature dependent mechanisms (Planel et al., 2004) as well as the hibernation-state dependent activity of tau kinases (Stieler et al., 2011) are responsible for the reversible tau phosphorylation that occurs during hibernation. Compared to other brain areas of the golden hamster the pattern and time course of tau phosphorylation in the olfactory bulb resembles closely the midbrain region as reported previously (Stieler et al., 2011). The only exception is the almost twofold expression of S396/S404 in the olfactory bulb, whereas in the other regions show only an increase up to 1.5 fold. Similar to basal forebrain and diagonal band (Härtig et al., 2007), phosphorylated tau protein was present in only some cell types while it was absent in others. In the periglomerular neurons of the olfactory bulb phosphorylated tau is present in some dopaminergic neurons, but is absent in GABAergic and acetylcholinergic neurons. There is no colocalization of hyperphosphorylated tau in cholinergic fibers and buttons in the external plexiform layer. Immature and mature granule cells, as identified by expression of doublecortin and calretinin, respectively, are devoid of phosphorylated tau. Phosphorylated tau is present in a few cell bodies located in the mitral cell layer and therefore we assume that the expression in the external plexiform layer belongs to the apical dendrites of mitral (and perhaps tufted cells). However, fluorescence double labeling using a suitable marker of mitral cells in adult Golden hamsters is necessary.

### Synaptic Plasticity

Spine density in Golgi-impregnated specimens (Popov and Bocharova, 1992; Popov et al., 1992, 2007; Magariños et al., 2006; Bullmann et al., 2016) and expression of synaptic proteins can be used to quantify the plastic changes in hibernation (Arendt et al., 2003; Strijkstra et al., 2003; von der Ohe et al., 2007; Bullmann et al., 2016). There is a single report (von der Ohe et al., 2007) using immunohistochemistry to show that plastic synaptic changes in hibernation are associated with a dissociation of proteins from the post-synaptic density rather than a degradation of these proteins. However, western blots that measure the total amount of proteins have shown changes in the expression of synaptic proteins during hibernation (Mirra et al., 1991; Härtig et al., 2007; Hoover et al., 2010) parallel to the spines regression (Popov and Bocharova, 1992; Magariños et al., 2006; Popov et al., 2007; Bullmann et al., 2016). Synaptic protein expression remained unchanged during hibernation as well as the spine density on olfactory bulb granule cells. This sparing of granule cell spines during hibernation was consistent with the absence of phosphorylated tau in olfactory bulb granule cells.

### Stability of Microtubules

Dendritic spines contain neurotransmitter receptors organized by specific scaffolding proteins, extensive actin cytoskeleton, which together with microtubules (Jaworski et al., 2009) regulates spine plasticity (Hotulainen and Hoogenraad, 2010). The microtubule binding protein tau stabilizes microtubules (Weingarten et al., 1975; Drechsel et al., 1992; Goode and Feinstein, 1994; Fryer et al., 1996; Prezel et al., 2018) and co-organizes dynamic microtubules and actin networks (Elie et al., 2015). Such cross-linking of microtubules and actin filaments depends on the phosphorylation of tau (Elie et al., 2015). Probably, tau phosphorylation during torpor uncouples microtubule in the spine neck and actin cytoskeleton in the spine head thus leading to spine retraction (Popov and Bocharova, 1992; Arendt et al., 2003; von der Ohe et al., 2006, 2007). Furthermore, its presence in post-synaptic spines is activity dependent (Frandemiche et al., 2014) and mediates the targeting of the src family kinase fyn to glutamatergic NMDA receptors (Ittner et al., 2010) and a reduction of tau expression results in synapse reduction (Chen et al., 2011). However, consistent with the constant synaptic protein expression and spine density in the olfactory bulb during hibernation we did not detect major changes of tubulin stability: This is also in agreement with previous reports (Yu et al., 1994; Planel et al., 2008) which indicated that tau does not markedly contribute to cold stability of microtubules. Tau phosphorylation and its subsequent detachment from microtubules during hibernation apparently have, thus, no influence on microtubule stability.

### Hibernation Might Impair Olfactory Memory

The effect of hibernation on olfactory discrimination was tested using an established two-odor discrimination task (Schellinck et al., 2001). After multiple exposures to a rewarded and a non-rewarded odor, animals were housed in a cold environment. When half of the animals went into hibernation, all animals were removed from the cold environment and tested for their odor preference. Hibernating animals did not show a significant preference for the odor that was previously presented with a food reward. On the other hand, non-hibernating animals showed a clear preference, which implies that they remembered the rewarded odor. Our study shows that this behavioral task originally described for mice can efficiently be used to assess olfactory learning and memory in hamsters. In the control group, olfactory memory traces last for almost 2 months, whereas the recall was impaired in the hibernation group (where animals experienced at least one torpor phase).

The observed impairment after hibernation could be explained in terms of sensation (reception), perception, memory and motivation. The odor sensation is the binding of the odor molecules to the receptor in the olfactory epithelium and the increased activity of a specific subtype of olfactory receptor neurons. Although we did not test for the sensation of odors, e.g., by calcium imaging of olfactory receptor responses (Ma and Shepherd, 2000) in hibernating animals, hematoxylin and eosin staining of the olfactory epithelium does not show any obvious damage after hibernation (not shown). The perception of odors takes place in the olfactory bulb; each type of receptor neurons projects to a different glomerulus and the discrimination between odor features is enhanced by lateral inhibition (Lledo and Lagier, 2006) of mitral cells mediated by the granule cells increase discrimination of odors (Mori et al., 1999). Mitral cells then project to principal cells in the piriform cortex (Knafo et al., 2001), which relay this olfactory information to the hippocampus (Hess et al., 1995; Knafo et al., 2004) as well as the prefrontal cortex (Alvarez and Eichenbaum, 2002). In turn granule and mitral cells are modulated by efferent inputs originating in central brain areas. It has been argued (Wilson et al., 2004), that peripheral olfactory system is more involved in implicit memory (e.g., perceptual learning) (Fletcher and Wilson, 2003) whereas the central olfactory system is encodes explicit memory (e.g., olfactory discrimination learning) (Eichenbaum et al., 1989; Alvarez and Eichenbaum, 2002). However, in the last 15 years it has become clear that adult born granule cells play a large role in olfactory discrimination learning. Olfactory discrimination learning specifically increases proximal spine density in adult born granule cells, their activity promotes odor-reward association while their immediate post-training ablation impair olfactory memory. Interestingly, ablation 28 days after training did not impair memory, indicating that long-term memory is independent of adult born granule cells (Arruda-Carvalho et al., 2014).

Previously we did not observed memory loss in a hippocampus-dependent task (Bullmann et al., 2016). Here we used the same reward (sunflower seeds) and the same hibernation paradigm. Therefore we do not expect that the impairment of odor discrimination is due to differences in motivation, as it has been argued before (McNamara and Riedesel, 1973; Alloway, 2008). We did not observe any difference in exploratory activity, further suggesting that the memory recall test was not affected by a change of motivation. As we summarized previously (Bullmann et al., 2016), the effect of hibernation on memory (McNamara and Riedesel, 1973; Millesi et al., 2001; Zhao et al., 2004; Clemens et al., 2009; Arendt and Bullmann, 2013; Bullmann et al., 2016) varies from species to species and depends on the testing paradigm. Therefore, we argue for species-specific differences in memory consolidation as required for specific behavior in the natural habitat. Previously, we hypothesized that the golden hamster must preserve spatial memory, but not social recognition. The Golden hamsters almost exclusively use olfactory cues processed by both main olfactory system (MOS) and the accessory or vomeronasal system (AOS) (Meredith, 1991) for regulating social interactions (Johnston, 1990; Petrulis, 2009). The impairment observed in the two-odor discrimination task is consistent with our hypothesis that during hibernation non-essential memory content may prone to erasure. Further behavioral experiments are needed to prove that this extends to impaired social kin recognition in Golden hamsters after arousal.

### Cholinergic Hypothesis of Odor Discrimination Impairment — Implications for Alzheimer’s Disease

Aggregated tau is present in the olfactory system in all definite Alzheimer’s disease cases and shows highly significant correlation with Braak staging in the brain (Attems et al., 2014). All layers are affected by the tau pathology and there are conflicting reports whether the dramatic loss of mitral cells is preceded by development of neurofibrillary tangles (Struble and Clark, 1992; Kovács et al., 1999). It has been argued that consequence of either tau pathology or mitral cell degeneration that leads to a dramatic loss of smell in Alzheimer’s disease might be a useful screening tool (Wesson et al., 2011; Attems et al., 2014). However, olfactory deficit may not be caused by tau phosphorylation, aggregation and cell death in the MOB, but by the diminished modulatory input, most important from acetylcholinergic neurons in the basal forebrain (Chaudhury et al., 2009; Gottfried, 2010; Ma and Luo, 2012; Devore et al., 2014; Rothermel et al., 2014). Cholinergic projection neurons in the basal forebrain can be classified according to their projection targets as Ch1–Ch4 regions (Mesulam et al., 1983). According to that classification, the lateral portion of the horizontal limb nucleus of the diagonal band (Ch3) provides the major cholinergic innervation to the olfactory bulb. Previously, we showed (Härtig et al., 2007) in the basal forebrain projection system of hibernating hamsters that cholinergic neurons are selectively affected by PHF-like phosphorylated tau, while γ-aminobutyric acid (GABA)ergic neurons are largely spared, similar to what has been observed in AD (Davies and Maloney, 1976; Whitehouse et al., 1981; Arendt et al., 1983; Härtig et al., 2002). Cognitive dysfunction in patients with AD correlates with both neuronal loss and tangle load in the basal forebrain (Holzer et al., 1994; Samuel et al., 1994; Iraizoz et al., 1999). It is possible that phosphorylation of tau in torpor is associated with a cholinergic deficit during the arousal phase. Such disruption cholinergic homoeostasis in nucleus basalis of Meynert and frontal cortex has been observed by activation of a major tau kinase, GSK3β (Wang et al., 2017). This might explain the observed impairment of olfactory discrimination in hibernation and perhaps in the very early, preclinical stages of Alzheimer’s disease (Attems and Jellinger, 2006; Attems et al., 2014; Roberts et al., 2016).

## Data Availability

The datasets generated for this study are available on request to the corresponding author.

## Ethics Statement

All experimental procedures on animals were carried out in accordance with the European Council Directive 86/609/EEC and 2010/63/EU to improve the welfare of animals used in scientific procedures and had been approved by the local authorities (T74/05, Regierungspräsidium Leipzig). Case recruitment, autopsy, and data handling have been performed in accordance with the ethical standards as laid down in the 1964 Declaration of Helsinki and its later amendments as well as with the convention of the Council of Europe on Human Rights and Biomedicine and had been approved by the responsible Ethics Committee of the Leipzig University.

## Author Contributions

TB designed the study, built the equipment, performed the experiments, analyzed the data, assembled the figures, interpreted the results, and prepared and revised the manuscript. EF performed the behavioral experiments, immunohistochemical staining, and spine measurements. TK performed the Western blotting and histochemical staining. VO performed the sampling of human olfactory bulb. MH and TA interpreted the results, and prepared and revised the manuscript.

## Conflict of Interest Statement

The authors declare that the research was conducted in the absence of any commercial or financial relationships that could be construed as a potential conflict of interest.

## Footnotes

1 http://www.R-project.org

2 http://r-forge.r-project.org/projects/lattice/

## Supplementary Material

FIGURE S1 High magnification images of the human olfactory bulb, showing the distribution of phosphorylated tau by AT8 immunohistochemistry and the distribution of tangles by standard Gallyas/Nissl stainind.

FIGURE S2 Habituation: The hamsters are initially placed in the middle of the three-chamber-apparatus for accomodation to the test procedure. No odors are present and they can freely explore the apparatus. Training: The hamsters are presented with an alternation of two odors, one of them paired with reward (sunflower seeds), the other without. They are given 4 trials each day, 4 times a week, for two weeks. They experience 16 CS+ and 16 CS- pairings in total. Testing: The hamsters are again placed in the middle of the three-chamber-apparatus, this time with both CS+ and CS- odors presented in the side chamber. This time, no food reward is given with CS+ odor.

TABLE S1 Raw data for the two odor discrimination task.
