# Supplementary material for: Hibernation Impairs Odor Discrimination – Implications for Alzheimer’s Disease
Source: Front Neuroanat. 2019 Jul 16;13:69. doi: 10.3389/fnana.2019.00069 (PMC6646461; doi:10.3389/fnana.2019.00069)

Supplemental Figure 1

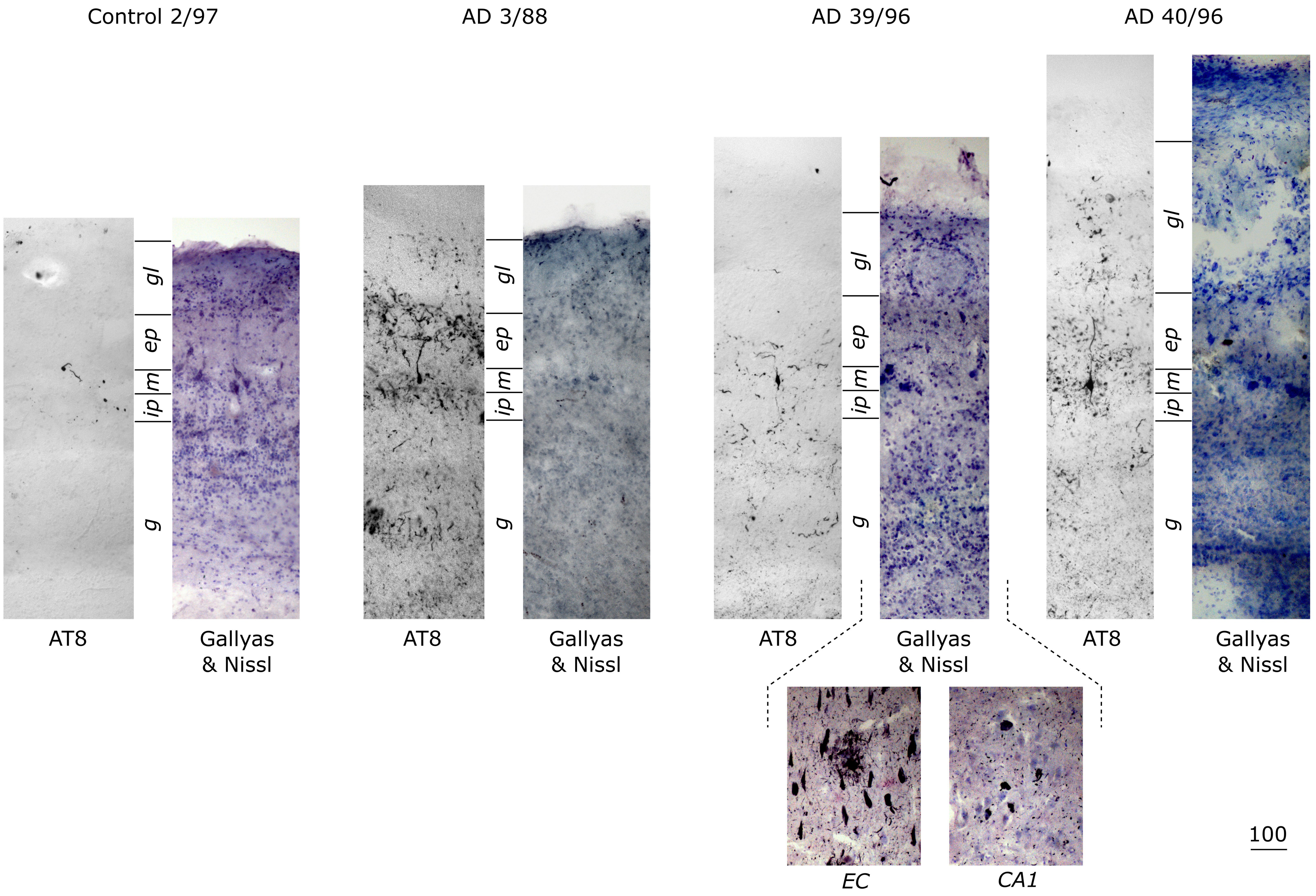

Supplement: FIGURE S1 — High magnification images of the human olfactory bulb, showing the distribution of phosphorylated tau by AT8 immunohistochemistry and the distribution of tangles by standard Gallyas/Nissl stainind. [file Data_Sheet_1.PDF]
