# Supplementary material for: Hibernation Impairs Odor Discrimination – Implications for Alzheimer’s Disease
Source: Front Neuroanat. 2019 Jul 16;13:69. doi: 10.3389/fnana.2019.00069 (PMC6646461; doi:10.3389/fnana.2019.00069)

Supplemental Figure 2

Habituation

The hamsters are initially placed in the middle of the three-chamber-apparatus for accomodation to the test procedure. No odors are present and they can freely explore the apparatus.

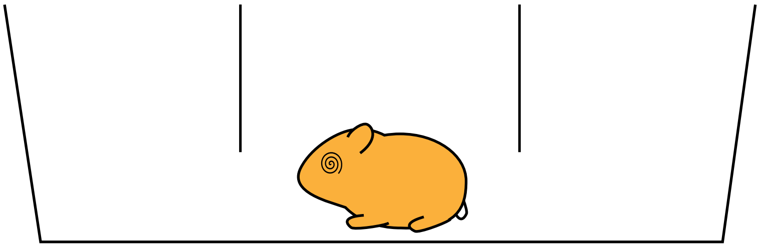

Training

The hamsters are presented with an alternation of two odors, one of them paired with reward (sunflower seeds), the other without. They are given 4 trials each day, 4 times a week, for two weeks. They experience 16 CS+ and 16 CS- pairings in total.

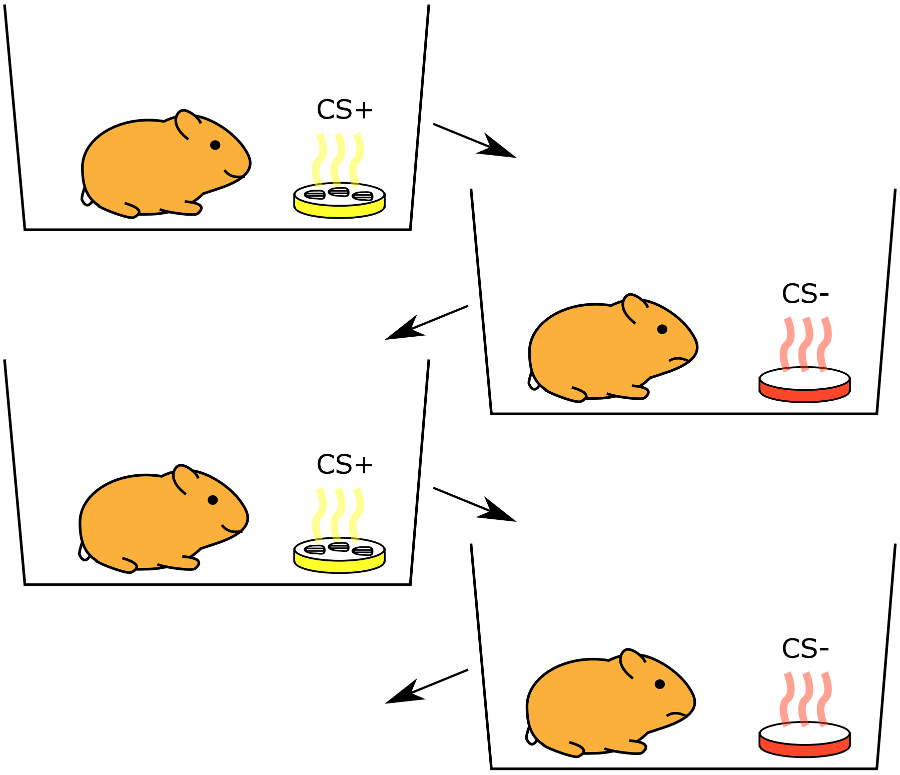

Testing

The hamsters are again placed in the middle of the three-chamber-apparatus, this time with both CS+ and CS- odors presented in the side chamber. This time, no food reward is given with CS+ odor.

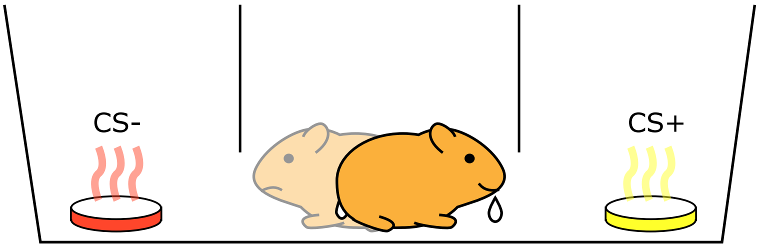

Supplement: FIGURE S2 — Habituation: The hamsters are initially placed in the middle of the three-chamber-apparatus for accomodation to the test procedure. No odors are present and they can freely explore the apparatus. Training: The hamsters are presented with an alternation of two odors, one of them paired with reward (sunflower seeds), the other without. They are given 4 trials each day, 4 times a week, for two weeks. They experience 16 CS+ and 16 CS- pairings in total. Testing: The hamsters are again placed in the middle of the three-chamber-apparatus, this time with both CS+ and CS- odors presented in the side chamber. This time, no food reward is given with CS+ odor. [file Data_Sheet_2.PDF]
